# 3α-Azido-5-cholestene

**DOI:** 10.1107/S1600536808025294

**Published:** 2008-08-09

**Authors:** Todd A. Houston, Sabina Quader, Sue E. Boyd, Ian D. Jenkins, Peter C. Healy

**Affiliations:** a Institute for Glycomics, Griffith University, Gold Coast 4222, Australia; b Eskitis Institute for Cell and Molecular Therapies, Griffith University, Brisbane 4111, Australia

## Abstract

The crystal structure of the title compound, C_27_H_45_N_3_, has been determined as part of our investigation into the hydro­phobic modification of amino­glycoside anti­biotics. The isopropyl group showed disorder for the tertiary carbon (equal occupancies), with high thermal motion for the peripheral atoms of the isopropyl and azide groups also apparent in the structure. The axial disposition of the azide group is consistent with the clean inversion of stereochemistry at C-3 under Mitsunobu conditions.

## Related literature

For related literature, see: Freiberg (1965 ▶); Loibner & Zbiral (1976 ▶); Quader *et al.* (2006 ▶, 2007 ▶); Stoffel & Klotzbuecher (1978 ▶); Viaud & Rollin (1990 ▶); Wilkinson *et al.* (2007 ▶).
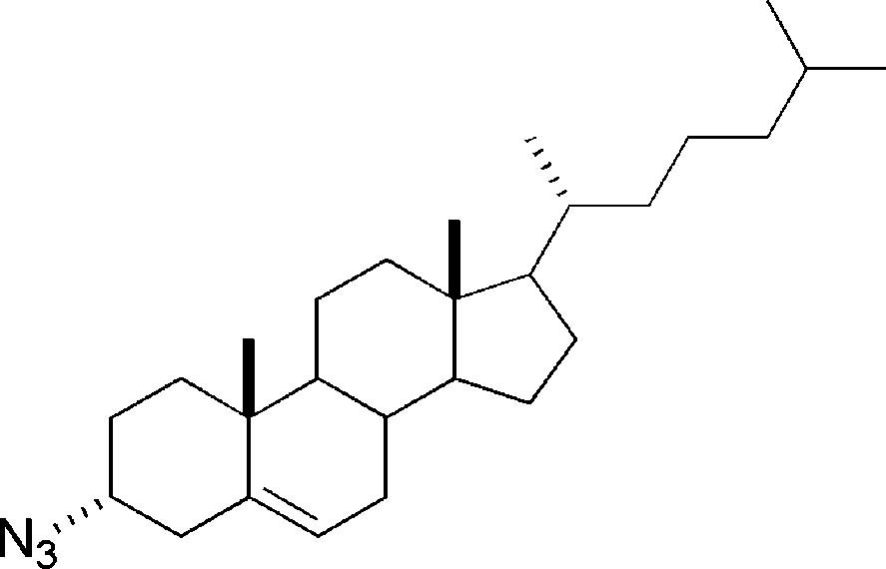


## Experimental

### 

#### Crystal data

C_27_H_45_N_3_
*M* *_r_* = 411.66
Monoclinic, 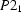

*a* = 13.3763 (3) Å
*b* = 6.2288 (1) Å
*c* = 15.0495 (4) Å
β = 94.205 (2)°
*V* = 1250.52 (5) Å^3^
*Z* = 2
Mo *K*α radiation
μ = 0.06 mm^−1^
*T* = 223 K
0.44 × 0.39 × 0.28 mm

#### Data collection

Oxford-Diffraction Gemini S Ultra diffractometer
Absorption correction: none
10298 measured reflections
2428 independent reflections
1883 reflections with *I* > 2σ(*I*)
*R*_int_ = 0.027

#### Refinement

*R*[*F* ^2^ > 2σ(*F* ^2^)] = 0.044
*wR*(*F* ^2^) = 0.125
*S* = 0.98
2428 reflections
280 parameters
1 restraint
H-atom parameters constrained
Δρ_max_ = 0.20 e Å^−3^
Δρ_min_ = −0.20 e Å^−3^

### 

Data collection: *CrysAlis CCD* (Oxford Diffraction, 2007 ▶); cell refinement: *CrysAlis RED* (Oxford Diffraction, 2007 ▶); data reduction: *CrysAlis RED*; program(s) used to solve structure: *SIR97* (Altomare *et al.*, 1999 ▶); program(s) used to refine structure: *SHELXL97* (Sheldrick, 2008 ▶); molecular graphics: *ORTEP-3 for Windows* (Farrugia, 1997 ▶); software used to prepare material for publication: *PLATON* (Spek, 2003 ▶).

## Supplementary Material

Crystal structure: contains datablocks global, I. DOI: 10.1107/S1600536808025294/tk2292sup1.cif

Structure factors: contains datablocks I. DOI: 10.1107/S1600536808025294/tk2292Isup2.hkl

Additional supplementary materials:  crystallographic information; 3D view; checkCIF report

## Figures and Tables

**Fig. 1.**
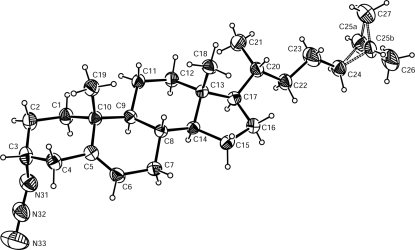
The molecular structure of (II), with atom labels and 40% probability displacement ellipsoids for non-H atoms.

**Fig. 2.**
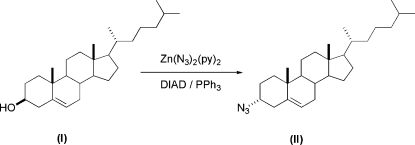
The formation of the title compound.

## supplementary crystallographic information

### Comment

As part of our program to identify new anti-tubercular compounds, we have created a diverse range of azide and alkyne coupling partners to generate a variety of compounds (Quader *et al.*, 2007; Wilkinson *et al.*, 2007). Among these are steroid and lipid components for the hydrophobic modification of aminoglycosides to aid in penetration of the waxy coat of mycobacteria (Quader *et al.*, 2006). Here, we describe the synthesis and X-ray crystal structure of 3α-azido-5-cholestene, (II, Fig. 1). This compound was previously tested for its ability to inhibit cholesterol biosynthesis (Stoffel *et al.*, 1978).

Compound (II) was synthesized directly from cholesterol (I) in 74% yield using the Mitsunobu reaction catalysed with the zinc azide-pyridine complex [Zn(N_3_)_2_(py)_2_] (Viaud & Rollin, 1990). This reaction compares favourably with the use of toxic HN_3_ for this purpose (Loibner & Zbiral, 1976). The clean inversion of stereochemistry under these conditions makes this method preferable in both efficiency and selectivity over azide displacement of 3-tosylate (Freiberg, 1965). A competing reaction at C-6 and a mixture of isomers at C-3 was reported in the latter case.

Compound (II) crystallizes in the polar space group *P*2_1_ with one molecule in the asymmetric unit.  The fused tetracyclic ring system adopts the expected conformations for the all-trans A/B/C/D junctions. The six-membered rings A and C adopt normal chair conformations. The C5—C6 bond length of 1.320 (4)Å confirms the presence of the double bond in this position while the axial disposition of the azide substituent on C3 confirms inversion of the alcohol stereochemistry has occurred. The azide group is almost linear with the N31—N32—N33 angle 173.4 (5)° while the C3—N31—N32 angle is 115.4 (4)°. The molecules are linked in the crystal lattice by van der Waals interactions only.

### Experimental

Diisopropyl azodicarboxylate (DIAD) (520 µ*L*, 2.58 mmol) was slowly added to a suspension of cholesterol (I) (500 mg, 1.29 mmol), triphenylphosphine (676 mg, 2.58 mmol) and [Zn(N_3_)_2_(py)_2_] (Viand & Rollin, 1990) (400 mg, 1.29 mmol) in toluene at RT and stirred (4 h). The reaction mixture was then concentrated under reduced pressure and subjected to column chromatography to furnish 3α-azido-5-cholestene as a pale-yellow solid (394 mg, 74%). Crystals suitable for X-ray diffraction analysis, were obtained after recrystallization from CDCl_3_: mp 387–389 K [lit. mp 388–389 K (Freiberg, 1965)].

^1^H NMR (400 MHz, CDCl_3_, 298 K): δ p.p.m. 5.41 (1*H*, m, H6), 3.88 (1*H*, dddd, J = 4, 5, 11, 11 Hz, H3), 2.54 (1*H*, ddd, J = 2.2, 2.2, 15 Hz H4a), 2.20 (1*H*, ddd, J = 2.5, 2.5, 15 Hz H4b), 2.06–1.91 (2*H*, m, H7a,16*a*), 1.90–0.97 (24*H*, m), 1.10 (3*H*, s, CH_3_,H19), 0.92 (3*H*, d, J = 6.6, CH_3_, H21), 0.88 (3*H*, d, J = 1.8, CH_3_, H26/H27) 0.86 (3*H*, s, J = 1.8, CH_3_, H26/H27) 0.69 (3*H*, s, CH_3_, H18).

^13^C{^1^H} NMR (100 MHz, CDCl_3_, 298 K): δ p.p.m. 138.06 (C5), 123.15 (C6), 58.26 (C3), 56.68 (C14), 56.10 (C17), 49.88 (C9), 42.28 (C13), 39.71 (C12), 39.51 (C24), 37.10 (C10), 36.18 (C22), 36.10 (C4), 35.80 (C20), 33.61(C1), 31.80, 31.76 (C7/C8), 28.22 (C16), 28.00 (C25), 26.10 (C2), 24.25 (C15), 23.81 (C23), 22.81, 22.55 (C26/27), 20.71 (C11), 18.98 (C19), 18.70 (C21), 11.84 (C18).

### Refinement

H atoms attached to carbon were constrained as riding atoms, with C–H set to 0.93 - 0.97Å. *U*_iso_(H) values were set to 1.2*U*_eq_ of the parent atom. The isopropyl group showed disorder with the C25 atom modelled as two atoms with 50% occupancy. High thermal motion of peripheral carbon atoms (C26, C27) for the isopropyl group and the nitrogen atom N33 of the azide group was also apparent in the structure. In the absence of significant anomalous dispersion effects, Friedel pairs were merged before refinement. The absolute configuration was assigned on the basis of the C atoms retaining their configuration during the synthesis of the azide.

### Crystal data

**Table:**

C_27_H_45_N_3_	*F*_000_ = 456
*M**_r_* = 411.66	*D*_x_ = 1.093 Mg m^−^^3^
Monoclinic, *P*2_1_	Mo *K*α radiation λ = 0.71070 Å
Hall symbol: P 2yb	Cell parameters from 6446 reflections
*a* = 13.3763 (3) Å	θ = 3.0–28.8º
*b* = 6.2288 (1) Å	µ = 0.06 mm^−^^1^
*c* = 15.0495 (4) Å	*T* = 223 K
β = 94.205 (2)º	Block, colourless
*V* = 1250.52 (5) Å^3^	0.44 × 0.39 × 0.28 mm
*Z* = 2	

### Data collection

**Table:**

Oxford-Diffraction GEMINI Ultra diffractometer	2428 independent reflections
Radiation source: Enhance (Mo) X-ray Source	1883 reflections with *I* > 2σ(*I*)
Monochromator: graphite	*R*_int_ = 0.027
Detector resolution: 16.0774 pixels mm^-1^	θ_max_ = 25.1º
*T* = 223 K	θ_min_ = 3.0º
ω and φ scans	*h* = 0→15
Absorption correction: none	*k* = −7→7
10298 measured reflections	*l* = −17→17

### Refinement

**Table:**

Refinement on *F*^2^	Secondary atom site location: difference Fourier map
Least-squares matrix: full	Hydrogen site location: inferred from neighbouring sites
*R*[*F*^2^ > 2σ(*F*^2^)] = 0.044	H-atom parameters constrained
*wR*(*F*^2^) = 0.125	*w* = 1/[σ^2^(*F*_o_^2^) + (0.0932*P*)^2^] where *P* = (*F*_o_^2^ + 2*F*_c_^2^)/3
*S* = 0.99	(Δ/σ)_max_ = 0.019
2428 reflections	Δρ_max_ = 0.20 e Å^−^^3^
280 parameters	Δρ_min_ = −0.20 e Å^−^^3^
1 restraint	Extinction correction: none
Primary atom site location: structure-invariant direct methods	

### Special details

**Table:**

Geometry. Bond distances, angles etc. have been calculated using the rounded fractional coordinates. All su's are estimated from the variances of the (full) variance-covariance matrix. The cell esds are taken into account in the estimation of distances, angles and torsion angles
Refinement. Refinement of *F*^2^ against ALL reflections. The weighted *R*-factor *wR* and goodness of fit *S* are based on *F*^2^, conventional *R*-factors *R* are based on *F*, with *F* set to zero for negative *F*^2^. The threshold expression of *F*^2^ > 2σ(*F*^2^) is used only for calculating *R*-factors(gt) *etc*. and is not relevant to the choice of reflections for refinement. *R*-factors based on *F*^2^ are statistically about twice as large as those based on *F*, and *R*- factors based on ALL data will be even larger.

### Fractional atomic coordinates and isotropic or equivalent isotropic displacement parameters (Å^2^)

**Table:**

	*x*	*y*	*z*	*U*_iso_*/*U*_eq_	Occ. (<1)
N31	0.0644 (2)	0.4907 (5)	−0.3662 (2)	0.0638 (11)	
N32	−0.0062 (3)	0.3729 (6)	−0.3817 (2)	0.0697 (12)	
N33	−0.0652 (3)	0.2474 (7)	−0.3963 (4)	0.124 (2)	
C1	0.2026 (2)	0.7988 (5)	−0.26902 (18)	0.0471 (10)	
C2	0.1368 (2)	0.8453 (6)	−0.35372 (19)	0.0529 (10)	
C3	0.0392 (2)	0.7231 (6)	−0.3553 (2)	0.0511 (10)	
C4	−0.0146 (2)	0.7594 (6)	−0.2716 (2)	0.0485 (10)	
C5	0.0524 (2)	0.7326 (5)	−0.18621 (18)	0.0390 (8)	
C6	0.0231 (2)	0.6140 (6)	−0.12045 (19)	0.0447 (9)	
C7	0.08021 (19)	0.5861 (5)	−0.03207 (18)	0.0422 (9)	
C8	0.16458 (19)	0.7473 (4)	−0.01577 (17)	0.0338 (8)	
C9	0.22078 (18)	0.7753 (4)	−0.10085 (17)	0.0350 (8)	
C10	0.1518 (2)	0.8525 (5)	−0.18214 (17)	0.0372 (8)	
C11	0.3143 (2)	0.9172 (5)	−0.08280 (18)	0.0448 (9)	
C12	0.38295 (19)	0.8491 (6)	−0.00183 (18)	0.0443 (9)	
C13	0.32634 (19)	0.8317 (5)	0.08311 (17)	0.0357 (8)	
C14	0.23823 (19)	0.6769 (4)	0.06031 (18)	0.0336 (8)	
C15	0.1992 (2)	0.6228 (5)	0.15046 (18)	0.0433 (9)	
C16	0.2943 (2)	0.6222 (5)	0.21424 (18)	0.0438 (9)	
C17	0.38102 (19)	0.7131 (5)	0.16239 (18)	0.0385 (8)	
C18	0.2909 (2)	1.0517 (5)	0.1121 (2)	0.0464 (9)	
C19	0.1302 (2)	1.0949 (6)	−0.1771 (2)	0.0512 (10)	
C20	0.4598 (2)	0.8319 (5)	0.22355 (18)	0.0447 (9)	
C21	0.5409 (3)	0.9376 (7)	0.1724 (2)	0.0687 (14)	
C22	0.5077 (2)	0.6834 (6)	0.2950 (2)	0.0504 (10)	
C23	0.5700 (3)	0.7965 (8)	0.3692 (3)	0.0881 (17)	
C24	0.6160 (2)	0.6573 (6)	0.4412 (2)	0.0546 (10)	
C25A	0.6989 (9)	0.746 (2)	0.5021 (7)	0.078 (4)	0.500
C25B	0.6538 (6)	0.7565 (18)	0.5303 (5)	0.052 (3)	0.500
C26	0.7182 (4)	0.6018 (10)	0.5855 (3)	0.1035 (19)	
C27	0.7052 (5)	0.9659 (11)	0.5206 (3)	0.113 (2)	
H1A	0.21890	0.64970	−0.26840	0.0570*	
H1B	0.26210	0.88060	−0.27010	0.0570*	
H2A	0.12310	0.99450	−0.35680	0.0640*	
H2B	0.17200	0.80390	−0.40390	0.0640*	
H3	−0.00290	0.76810	−0.40540	0.0610*	
H4A	−0.04100	0.90040	−0.27320	0.0580*	
H4B	−0.06800	0.65840	−0.27090	0.0580*	
H6	−0.03910	0.53940	−0.12970	0.0530*	
H7A	0.10770	0.44550	−0.02920	0.0500*	
H7B	0.03490	0.60240	0.01340	0.0500*	
H8	0.13670	0.88140	−0.00080	0.0410*	
H9	0.24440	0.63670	−0.11570	0.0420*	
H11A	0.29270	1.05990	−0.07360	0.0540*	
H11B	0.35200	0.91200	−0.13390	0.0540*	
H12A	0.41150	0.71340	−0.01370	0.0530*	
H12B	0.43470	0.95260	0.00770	0.0530*	
H14	0.26750	0.54780	0.04050	0.0400*	
H15A	0.16750	0.48620	0.14910	0.0520*	
H15B	0.15320	0.72890	0.16750	0.0520*	
H16A	0.30940	0.47920	0.23310	0.0530*	
H16B	0.28460	0.70910	0.26450	0.0530*	
H17	0.41400	0.59300	0.13840	0.0460*	
H18A	0.34760	1.13990	0.12730	0.0560*	
H18B	0.25080	1.11550	0.06480	0.0560*	
H18C	0.25290	1.03570	0.16260	0.0560*	
H19A	0.07130	1.12730	−0.21380	0.0610*	
H19B	0.18520	1.17230	−0.19740	0.0610*	
H19C	0.12080	1.13310	−0.11730	0.0610*	
H20	0.42610	0.94200	0.25290	0.0540*	
H21A	0.52340	1.08250	0.16040	0.0830*	
H21B	0.54670	0.86300	0.11780	0.0830*	
H21C	0.60310	0.93140	0.20700	0.0830*	
H22A	0.54990	0.58520	0.26700	0.0610*	
H22B	0.45570	0.60650	0.32070	0.0610*	
H23A	0.52790	0.89640	0.39580	0.1060*	
H23B	0.62260	0.87000	0.34320	0.1060*	
H24A	0.67160	0.58730	0.41790	0.0660*	
H24B	0.56710	0.55250	0.45380	0.0660*	
H25A	0.75650	0.72340	0.46960	0.0950*	0.500
H25B	0.59740	0.78220	0.56240	0.0560*	0.500
H26A	0.78450	0.61280	0.56720	0.1210*	
H26B	0.72010	0.63670	0.64560	0.1210*	
H26C	0.69710	0.45970	0.57450	0.1210*	
H27A	0.66620	1.05800	0.48200	0.1360*	
H27B	0.76900	0.94260	0.49580	0.1360*	
H27C	0.71770	1.03240	0.57720	0.1360*	

### Atomic displacement parameters (Å^2^)

**Table:**

	*U*^11^	*U*^22^	*U*^33^	*U*^12^	*U*^13^	*U*^23^
N31	0.0661 (17)	0.0602 (19)	0.0640 (19)	0.0036 (18)	−0.0020 (15)	−0.0113 (16)
N32	0.079 (2)	0.058 (2)	0.068 (2)	0.009 (2)	−0.0227 (17)	0.0007 (16)
N33	0.106 (3)	0.067 (2)	0.187 (5)	−0.016 (3)	−0.062 (3)	0.006 (3)
C1	0.0395 (14)	0.063 (2)	0.0391 (15)	−0.0020 (15)	0.0051 (12)	0.0025 (15)
C2	0.0502 (17)	0.069 (2)	0.0391 (15)	0.0002 (18)	0.0008 (13)	0.0010 (17)
C3	0.0527 (18)	0.059 (2)	0.0399 (16)	0.0085 (17)	−0.0079 (14)	0.0024 (16)
C4	0.0409 (14)	0.0579 (19)	0.0457 (16)	0.0007 (15)	−0.0040 (13)	−0.0040 (16)
C5	0.0351 (13)	0.0414 (16)	0.0406 (15)	0.0036 (14)	0.0033 (11)	−0.0027 (14)
C6	0.0377 (14)	0.0528 (18)	0.0432 (15)	−0.0075 (15)	0.0008 (12)	−0.0061 (15)
C7	0.0391 (14)	0.0449 (17)	0.0427 (15)	−0.0069 (14)	0.0042 (12)	−0.0001 (15)
C8	0.0344 (13)	0.0327 (13)	0.0341 (13)	−0.0012 (12)	0.0016 (11)	−0.0005 (12)
C9	0.0334 (13)	0.0375 (16)	0.0343 (13)	0.0013 (12)	0.0041 (11)	−0.0041 (12)
C10	0.0375 (14)	0.0416 (16)	0.0328 (14)	−0.0005 (13)	0.0046 (12)	−0.0009 (13)
C11	0.0432 (15)	0.0564 (19)	0.0349 (15)	−0.0091 (15)	0.0042 (12)	0.0051 (14)
C12	0.0348 (13)	0.0584 (18)	0.0397 (15)	−0.0070 (15)	0.0028 (12)	0.0005 (15)
C13	0.0370 (13)	0.0366 (15)	0.0332 (13)	−0.0031 (13)	0.0012 (11)	0.0009 (12)
C14	0.0373 (13)	0.0277 (14)	0.0360 (13)	0.0022 (12)	0.0050 (11)	0.0002 (11)
C15	0.0455 (15)	0.0436 (16)	0.0414 (15)	−0.0044 (15)	0.0073 (12)	0.0059 (14)
C16	0.0487 (15)	0.0456 (17)	0.0369 (15)	−0.0012 (15)	0.0021 (12)	0.0014 (14)
C17	0.0407 (14)	0.0349 (15)	0.0396 (14)	0.0044 (13)	0.0016 (12)	0.0001 (13)
C18	0.0542 (17)	0.0362 (16)	0.0476 (16)	−0.0009 (14)	−0.0035 (14)	−0.0020 (14)
C19	0.0595 (18)	0.0455 (17)	0.0473 (17)	0.0024 (17)	−0.0049 (14)	0.0036 (16)
C20	0.0442 (15)	0.0504 (17)	0.0390 (15)	−0.0019 (15)	−0.0009 (12)	−0.0019 (15)
C21	0.0580 (19)	0.089 (3)	0.057 (2)	−0.024 (2)	−0.0108 (16)	0.016 (2)
C22	0.0465 (16)	0.062 (2)	0.0416 (15)	−0.0025 (16)	−0.0034 (13)	0.0008 (15)
C23	0.107 (3)	0.078 (3)	0.072 (3)	−0.003 (3)	−0.042 (2)	0.004 (2)
C24	0.0471 (16)	0.068 (2)	0.0470 (17)	−0.0042 (17)	−0.0075 (14)	0.0016 (16)
C25A	0.081 (7)	0.086 (7)	0.063 (6)	0.010 (7)	−0.024 (5)	−0.005 (6)
C25B	0.052 (5)	0.070 (5)	0.032 (4)	−0.002 (5)	−0.001 (3)	−0.005 (4)
C26	0.129 (4)	0.106 (3)	0.067 (3)	−0.003 (4)	−0.051 (3)	0.007 (3)
C27	0.141 (4)	0.121 (5)	0.073 (3)	−0.052 (4)	−0.020 (3)	−0.008 (3)

### Geometric parameters (Å, °)

**Table:**

N31—N32	1.205 (5)	C4—H4A	0.9500
N31—C3	1.498 (5)	C4—H4B	0.9500
N32—N33	1.121 (6)	C6—H6	0.9500
C1—C2	1.523 (4)	C7—H7A	0.9500
C1—C10	1.554 (4)	C7—H7B	0.9500
C2—C3	1.510 (4)	C8—H8	0.9500
C3—C4	1.513 (4)	C9—H9	0.9500
C4—C5	1.521 (4)	C11—H11A	0.9500
C5—C6	1.317 (4)	C11—H11B	0.9500
C5—C10	1.522 (4)	C12—H12A	0.9500
C6—C7	1.495 (4)	C12—H12B	0.9500
C7—C8	1.517 (4)	C14—H14	0.9500
C8—C9	1.542 (4)	C15—H15A	0.9500
C8—C14	1.519 (4)	C15—H15B	0.9500
C9—C10	1.554 (4)	C16—H16A	0.9500
C9—C11	1.540 (4)	C16—H16B	0.9500
C10—C19	1.540 (5)	C17—H17	0.9500
C11—C12	1.531 (4)	C18—H18A	0.9500
C12—C13	1.537 (4)	C18—H18B	0.9500
C13—C14	1.542 (4)	C18—H18C	0.9500
C13—C17	1.542 (4)	C19—H19A	0.9500
C13—C18	1.524 (4)	C19—H19B	0.9500
C14—C15	1.527 (4)	C19—H19C	0.9500
C15—C16	1.536 (4)	C20—H20	0.9500
C16—C17	1.552 (4)	C21—H21A	0.9500
C17—C20	1.537 (4)	C21—H21B	0.9500
C20—C21	1.525 (5)	C21—H21C	0.9500
C20—C22	1.524 (4)	C22—H22A	0.9500
C22—C23	1.517 (5)	C22—H22B	0.9500
C23—C24	1.486 (6)	C23—H23A	0.9500
C24—C25A	1.492 (12)	C23—H23B	0.9500
C24—C25B	1.528 (9)	C24—H24A	0.9500
C25A—C25B	0.765 (14)	C24—H24B	0.9500
C25A—C26	1.550 (12)	C25A—H25A	0.9500
C25A—C27	1.399 (14)	C25B—H25B	0.9400
C25B—C26	1.502 (11)	C26—H26A	0.9500
C25B—C27	1.487 (13)	C26—H26B	0.9300
C1—H1A	0.9500	C26—H26C	0.9400
C1—H1B	0.9500	C27—H27A	0.9500
C2—H2A	0.9500	C27—H27B	0.9700
C2—H2B	0.9500	C27—H27C	0.9500
C3—H3	0.9500		
			
N31···H1A	2.6400	H12A···H21A^v^	2.5700
N32···H2A^i^	2.9300	H12B···C21	2.7700
N32···H4B	2.6100	H12B···H18A	2.5000
N33···H4A^i^	2.8500	H12B···H21B	2.2200
C12···C21	3.291 (4)	H14···C11	3.0500
C18···C21	3.474 (5)	H14···H7A	2.4000
C21···C12	3.291 (4)	H14···H9	2.4100
C21···C18	3.474 (5)	H14···H12A	2.3800
C1···H11B	2.8300	H14···H17	2.3800
C2···H19A	2.9300	H15A···C7	2.9500
C4···H19A	2.6800	H15B···C18	2.8900
C5···H8	3.0700	H15B···H18C	2.3300
C6···H9	2.9600	H15B···H6^vi^	2.5000
C7···H15A	2.9500	H16A···C22	3.0300
C8···H18B	2.8000	H16A···H22B	2.4100
C8···H19C	2.8800	H16B···C22	2.9900
C11···H1B	2.8600	H16B···H18C	2.5600
C11···H19C	2.9300	H16B···H20	2.4000
C11···H19B	2.8400	H16B···H22B	2.4700
C11···H14	3.0500	H17···H12A	2.4100
C11···H18B	2.7300	H17···H14	2.3800
C12···H21B	2.7300	H17···H21B	2.4800
C13···H21B	2.9600	H17···H22A	2.5600
C15···H7B	2.9000	H18A···C20	2.7800
C15···H18C	2.6700	H18A···C21	2.9100
C15···H7A	3.0900	H18A···H12B	2.5000
C16···H22B	2.5900	H18A···H20	2.4300
C16···H18C	2.7400	H18A···H21A	2.3900
C18···H11A	2.8000	H18B···C8	2.8000
C18···H8	2.7800	H18B···C11	2.7300
C18···H20	2.7700	H18B···H8	2.2800
C18···H15B	2.8900	H18B···H11A	2.2200
C19···H4A	2.8800	H18C···C15	2.6700
C19···H2A	2.7700	H18C···C16	2.7400
C19···H8	2.9600	H18C···H15B	2.3300
C19···H11A	2.5900	H18C···H16B	2.5600
C20···H18A	2.7800	H19A···C2	2.9300
C21···H12B	2.7700	H19A···C4	2.6800
C21···H18A	2.9100	H19A···H2A	2.4500
C21···H23B	2.7500	H19A···H4A	2.2000
C21···H12A^ii^	3.0500	H19B···C11	2.8400
C22···H16A	3.0300	H19B···H1B	2.3900
C22···H16B	2.9900	H19B···H11A	2.3700
C23···H21C	2.6500	H19C···C8	2.8800
C23···H27A	2.6200	H19C···C11	2.9300
C27···H23A	2.9500	H19C···H7A^iii^	2.3700
C27···H23B	2.8800	H19C···H8	2.3500
H1A···N31	2.6400	H19C···H11A	2.3900
H1A···H9	2.3000	H20···C18	2.7700
H1B···C11	2.8600	H20···H16B	2.4000
H1B···H11B	2.3100	H20···H18A	2.4300
H1B···H19B	2.3900	H20···H23A	2.4800
H2A···N32^iii^	2.9300	H21A···H18A	2.3900
H2A···C19	2.7700	H21A···H12A^ii^	2.5700
H2A···H19A	2.4500	H21B···C12	2.7300
H4A···N33^iii^	2.8500	H21B···C13	2.9600
H4A···C19	2.8800	H21B···H12B	2.2200
H4A···H19A	2.2000	H21B···H17	2.4800
H4B···N32	2.6100	H21C···C23	2.6500
H4B···H6	2.2600	H21C···H22A	2.4600
H6···H4B	2.2600	H21C···H23B	2.0800
H6···H15B^iv^	2.5000	H22A···H17	2.5600
H7A···C15	3.0900	H22A···H21C	2.4600
H7A···H14	2.4000	H22B···C16	2.5900
H7A···H19C^i^	2.3700	H22B···H16A	2.4100
H7B···C15	2.9000	H22B···H16B	2.4700
H8···C5	3.0700	H22B···H24B	2.4300
H8···C18	2.7800	H23A···C27	2.9500
H8···C19	2.9600	H23A···H20	2.4800
H8···H18B	2.2800	H23A···H27A	2.4000
H8···H19C	2.3500	H23B···C21	2.7500
H9···C6	2.9600	H23B···C27	2.8800
H9···H1A	2.3000	H23B···H21C	2.0800
H9···H14	2.4100	H23B···H27A	2.4300
H11A···C18	2.8000	H24A···H26C	2.4900
H11A···C19	2.5900	H24B···H22B	2.4300
H11A···H18B	2.2200	H24B···H26C	2.4900
H11A···H19B	2.3700	H26A···H27B	2.3200
H11A···H19C	2.3900	H26C···H24A	2.4900
H11B···C1	2.8300	H26C···H24B	2.4900
H11B···H1B	2.3100	H27A···C23	2.6200
H12A···H14	2.3800	H27A···H23A	2.4000
H12A···H17	2.4100	H27A···H23B	2.4300
H12A···C21^v^	3.0500	H27B···H26A	2.3200
			
N32—N31—C3	115.5 (3)	C10—C9—H9	107.00
N31—N32—N33	173.2 (5)	C11—C9—H9	106.00
C2—C1—C10	113.7 (2)	C9—C11—H11A	108.00
C1—C2—C3	111.3 (3)	C9—C11—H11B	108.00
N31—C3—C2	106.7 (2)	C12—C11—H11A	108.00
N31—C3—C4	111.1 (3)	C12—C11—H11B	108.00
C2—C3—C4	112.0 (3)	H11A—C11—H11B	110.00
C3—C4—C5	113.6 (2)	C11—C12—H12A	109.00
C4—C5—C6	120.5 (3)	C11—C12—H12B	109.00
C4—C5—C10	116.0 (2)	C13—C12—H12A	109.00
C6—C5—C10	123.5 (2)	C13—C12—H12B	109.00
C5—C6—C7	125.0 (3)	H12A—C12—H12B	110.00
C6—C7—C8	113.1 (2)	C8—C14—H14	105.00
C7—C8—C9	110.1 (2)	C13—C14—H14	106.00
C7—C8—C14	111.4 (2)	C15—C14—H14	105.00
C9—C8—C14	109.3 (2)	C14—C15—H15A	111.00
C8—C9—C10	113.0 (2)	C14—C15—H15B	111.00
C8—C9—C11	111.0 (2)	C16—C15—H15A	111.00
C10—C9—C11	112.9 (2)	C16—C15—H15B	111.00
C1—C10—C5	107.2 (2)	H15A—C15—H15B	109.00
C1—C10—C9	109.0 (2)	C15—C16—H16A	110.00
C1—C10—C19	110.4 (2)	C15—C16—H16B	110.00
C5—C10—C9	110.3 (2)	C17—C16—H16A	110.00
C5—C10—C19	108.5 (2)	C17—C16—H16B	110.00
C9—C10—C19	111.6 (2)	H16A—C16—H16B	110.00
C9—C11—C12	114.4 (2)	C13—C17—H17	107.00
C11—C12—C13	112.2 (2)	C16—C17—H17	107.00
C12—C13—C14	105.9 (2)	C20—C17—H17	107.00
C12—C13—C17	116.3 (2)	C13—C18—H18A	109.00
C12—C13—C18	110.9 (3)	C13—C18—H18B	109.00
C14—C13—C17	100.6 (2)	C13—C18—H18C	109.00
C14—C13—C18	112.2 (2)	H18A—C18—H18B	110.00
C17—C13—C18	110.4 (2)	H18A—C18—H18C	109.00
C8—C14—C13	115.5 (2)	H18B—C18—H18C	110.00
C8—C14—C15	119.2 (2)	C10—C19—H19A	109.00
C13—C14—C15	104.2 (2)	C10—C19—H19B	109.00
C14—C15—C16	103.6 (2)	C10—C19—H19C	109.00
C15—C16—C17	107.5 (2)	H19A—C19—H19B	110.00
C13—C17—C16	103.6 (2)	H19A—C19—H19C	110.00
C13—C17—C20	119.9 (3)	H19B—C19—H19C	110.00
C16—C17—C20	112.3 (2)	C17—C20—H20	107.00
C17—C20—C21	112.8 (2)	C21—C20—H20	108.00
C17—C20—C22	111.4 (3)	C22—C20—H20	107.00
C21—C20—C22	109.9 (2)	C20—C21—H21A	109.00
C20—C22—C23	114.7 (3)	C20—C21—H21B	109.00
C22—C23—C24	116.2 (4)	C20—C21—H21C	109.00
C23—C24—C25A	118.8 (5)	H21A—C21—H21B	110.00
C23—C24—C25B	119.8 (5)	H21A—C21—H21C	110.00
C25A—C24—C25B	29.3 (5)	H21B—C21—H21C	109.00
C24—C25A—C25B	78.0 (11)	C20—C22—H22A	108.00
C24—C25A—C26	110.8 (8)	C20—C22—H22B	108.00
C24—C25A—C27	121.1 (9)	C23—C22—H22A	108.00
C25B—C25A—C26	72.0 (11)	C23—C22—H22B	108.00
C25B—C25A—C27	81.1 (13)	H22A—C22—H22B	109.00
C26—C25A—C27	113.7 (8)	C22—C23—H23A	108.00
C24—C25B—C25A	72.7 (10)	C22—C23—H23B	108.00
C24—C25B—C26	111.4 (7)	C24—C23—H23A	108.00
C24—C25B—C27	113.2 (6)	C24—C23—H23B	108.00
C25A—C25B—C26	79.0 (11)	H23A—C23—H23B	110.00
C25A—C25B—C27	68.4 (12)	C23—C24—H24A	107.00
C26—C25B—C27	111.5 (6)	C23—C24—H24B	107.00
C25A—C26—C25B	29.0 (5)	C25A—C24—H24A	80.00
C25A—C27—C25B	30.6 (6)	C25A—C24—H24B	128.00
C2—C1—H1A	108.00	C25B—C24—H24A	107.00
C2—C1—H1B	109.00	C25B—C24—H24B	107.00
C10—C1—H1A	108.00	H24A—C24—H24B	109.00
C10—C1—H1B	109.00	C24—C25A—H25A	103.00
H1A—C1—H1B	109.00	C25B—C25A—H25A	176.00
C1—C2—H2A	109.00	C26—C25A—H25A	104.00
C1—C2—H2B	109.00	C27—C25A—H25A	102.00
C3—C2—H2A	109.00	C24—C25B—H25B	107.00
C3—C2—H2B	109.00	C25A—C25B—H25B	175.00
H2A—C2—H2B	109.00	C26—C25B—H25B	106.00
N31—C3—H3	109.00	C27—C25B—H25B	107.00
C2—C3—H3	109.00	C25A—C26—H26A	80.00
C4—C3—H3	109.00	C25A—C26—H26B	130.00
C3—C4—H4A	108.00	C25A—C26—H26C	112.00
C3—C4—H4B	108.00	C25B—C26—H26A	108.00
C5—C4—H4A	109.00	C25B—C26—H26B	111.00
C5—C4—H4B	108.00	C25B—C26—H26C	111.00
H4A—C4—H4B	110.00	H26A—C26—H26B	108.00
C5—C6—H6	118.00	H26A—C26—H26C	107.00
C7—C6—H6	117.00	H26B—C26—H26C	112.00
C6—C7—H7A	109.00	C25A—C27—H27A	117.00
C6—C7—H7B	108.00	C25A—C27—H27B	80.00
C8—C7—H7A	109.00	C25A—C27—H27C	128.00
C8—C7—H7B	109.00	C25B—C27—H27A	111.00
H7A—C7—H7B	109.00	C25B—C27—H27B	109.00
C7—C8—H8	109.00	C25B—C27—H27C	110.00
C9—C8—H8	109.00	H27A—C27—H27B	108.00
C14—C8—H8	109.00	H27A—C27—H27C	110.00
C8—C9—H9	106.00	H27B—C27—H27C	108.00
			
N32—N31—C3—C2	−169.7 (3)	C18—C13—C14—C8	61.1 (3)
N32—N31—C3—C4	67.9 (4)	C18—C13—C14—C15	−71.7 (3)
C10—C1—C2—C3	−57.8 (4)	C12—C13—C17—C16	−152.4 (3)
C2—C1—C10—C5	54.5 (3)	C12—C13—C17—C20	81.5 (3)
C2—C1—C10—C9	173.8 (2)	C14—C13—C17—C16	−38.6 (3)
C2—C1—C10—C19	−63.4 (3)	C14—C13—C17—C20	−164.7 (2)
C1—C2—C3—N31	−69.2 (3)	C18—C13—C17—C16	80.1 (3)
C1—C2—C3—C4	52.7 (4)	C18—C13—C17—C20	−46.0 (3)
N31—C3—C4—C5	70.8 (3)	C8—C14—C15—C16	−164.8 (2)
C2—C3—C4—C5	−48.5 (4)	C13—C14—C15—C16	−34.2 (3)
C3—C4—C5—C6	−131.6 (3)	C14—C15—C16—C17	9.5 (3)
C3—C4—C5—C10	49.4 (4)	C15—C16—C17—C13	18.5 (3)
C4—C5—C6—C7	−176.6 (3)	C15—C16—C17—C20	149.3 (2)
C10—C5—C6—C7	2.4 (5)	C13—C17—C20—C21	−53.5 (4)
C4—C5—C10—C1	−50.2 (3)	C13—C17—C20—C22	−177.6 (2)
C4—C5—C10—C9	−168.6 (2)	C16—C17—C20—C21	−175.3 (3)
C4—C5—C10—C19	69.0 (3)	C16—C17—C20—C22	60.6 (3)
C6—C5—C10—C1	130.9 (3)	C17—C20—C22—C23	−168.0 (3)
C6—C5—C10—C9	12.4 (4)	C21—C20—C22—C23	66.3 (3)
C6—C5—C10—C19	−110.0 (3)	C20—C22—C23—C24	178.7 (3)
C5—C6—C7—C8	13.4 (4)	C22—C23—C24—C25A	163.8 (6)
C6—C7—C8—C9	−42.4 (3)	C22—C23—C24—C25B	−162.5 (4)
C6—C7—C8—C14	−163.7 (2)	C23—C24—C25A—C25B	100.3 (12)
C7—C8—C9—C10	58.7 (3)	C23—C24—C25A—C26	165.6 (5)
C7—C8—C9—C11	−173.2 (2)	C23—C24—C25A—C27	28.6 (11)
C14—C8—C9—C10	−178.7 (2)	C25B—C24—C25A—C26	65.3 (11)
C14—C8—C9—C11	−50.7 (3)	C25B—C24—C25A—C27	−71.7 (13)
C7—C8—C14—C13	−179.2 (2)	C23—C24—C25B—C25A	−96.5 (12)
C7—C8—C14—C15	−53.9 (3)	C23—C24—C25B—C26	−166.8 (4)
C9—C8—C14—C13	59.0 (3)	C23—C24—C25B—C27	−40.2 (8)
C9—C8—C14—C15	−175.7 (2)	C25A—C24—C25B—C26	−70.3 (12)
C8—C9—C10—C1	−160.0 (2)	C25A—C24—C25B—C27	56.4 (11)
C8—C9—C10—C5	−42.6 (3)	C24—C25A—C25B—C26	116.8 (6)
C8—C9—C10—C19	78.0 (3)	C24—C25A—C25B—C27	−124.6 (6)
C11—C9—C10—C1	73.0 (3)	C26—C25A—C25B—C24	−116.8 (6)
C11—C9—C10—C5	−169.6 (2)	C26—C25A—C25B—C27	118.6 (6)
C11—C9—C10—C19	−49.0 (3)	C27—C25A—C25B—C24	124.6 (6)
C8—C9—C11—C12	50.6 (3)	C27—C25A—C25B—C26	−118.6 (6)
C10—C9—C11—C12	178.7 (2)	C24—C25A—C26—C25B	−69.1 (12)
C9—C11—C12—C13	−54.1 (4)	C27—C25A—C26—C25B	71.3 (12)
C11—C12—C13—C14	54.9 (3)	C24—C25A—C27—C25B	70.1 (11)
C11—C12—C13—C17	165.7 (3)	C26—C25A—C27—C25B	−65.8 (10)
C11—C12—C13—C18	−67.0 (3)	C24—C25B—C26—C25A	66.3 (11)
C12—C13—C14—C8	−60.1 (3)	C27—C25B—C26—C25A	−61.3 (11)
C12—C13—C14—C15	167.2 (2)	C24—C25B—C27—C25A	−58.8 (10)
C17—C13—C14—C8	178.5 (2)	C26—C25B—C27—C25A	67.8 (11)
C17—C13—C14—C15	45.7 (2)		

Symmetry codes: (i) *x*, *y*−1, *z*; (ii) −*x*+1, *y*+1/2, −*z*; (iii) *x*, *y*+1, *z*; (iv) −*x*, *y*−1/2, −*z*; (v) −*x*+1, *y*−1/2, −*z*; (vi) −*x*, *y*+1/2, −*z*.

### Hydrogen-bond geometry (Å, °)

**Table:**

*D*—H···*A*	*D*—H	H···*A*	*D*···*A*	*D*—H···*A*
C4—H4B···N32	0.95	2.61	2.929 (5)	100
